# PIWI pathway: bridging acute myeloid leukemia stemness and cellular differentiation

**DOI:** 10.3389/fcell.2024.1449353

**Published:** 2024-08-12

**Authors:** M. R. Garcia-Silva, M. E. Márquez, N. Pinello

**Affiliations:** Functional Genomics Laboratory, Institut Pasteur Montevideo, Montevideo, Uruguay

**Keywords:** PIWI, piwi associated RNA, AML, monocytes, macrophage

## Abstract

PIWI proteins are stem cell-associated RNA-binding proteins crucial for survival of germ stem cells. In cancer, PIWI proteins are overexpressed. Specifically, PIWIL4 is highly expressed in multiple cancers with the highest levels found in acute myeloid leukemia (AML), an aggressive malignancy propagated by a population of leukemia stem cells (LSCs). Bamezai et al. (Blood Journal, blood, 2023, 142, 90–105) demonstrated that PIWIL4 supports AML blasts and LSCs but is not necessary for healthy human hematopoietic progenitor stem cells (HSPCs) function *in vivo*. PIWIL4 in AML acts by preventing the accumulation of R-loops in key genes for LSCs persistence implicated in: DNA damage, replicative stress, and transcription arrest. We report that PIWIL4 expression significantly decreases in THP-1 monocytes exposed to a differentiating agent, suggesting a potential role for PIWIL4 in maintaining the undifferentiated state of myeloid cells. PIWIL4 overexpression could lead to the emergence of LSCs, driving leukemia propagation and maintenance. Our findings correlate with the persistent overexpression of PIWIL4 in myeloid cancers as reported by Bamezai et al., and suggest that PIWIL4 may be involved in myeloid cell differentiation. In this perspective, we highlight recent findings on the implication of PIWI pathway in maintaining AML stemness. Additionally, we propose further investigation on the role of PIWI pathway in oncogenesis and cellular differentiation as a strategy to identify biomarkers and therapeutic targets for AML.

## 1 Introduction

### 1.1 PIWI proteins and cancer

Since it was first described, the machinery of small non-coding RNAs (sncRNAs) has emerged as a new class of endogenous regulators of gene expression and genome integrity. sncRNAs are involved in key cellular processes such as cell differentiation, growth/proliferation, migration, apoptosis/death, metabolism and genome protection. The most well-known members of this family are micro RNAs (miRNAs), small interfering (siRNAs), PIWI-interacting RNAs (piRNAs) and transference RNA-derived fragments (Garcia-Silva et al., 2012).

PIWI proteins (P-element Induced WImpy testis Proteins) bind specific RNAs called PIWI-interacting RNAs (piRNAs). The PIWI-piRNAs pathway has highly conserved roles in germline development, gametogenesis, transposon silencing, and epigenetic regulation (Aravin et al., 2006; Girard et al., 2006; Brennecke et al., 2007; Lin, 2007). Furthermore, PIWI subfamily proteins are known by their highly conserved role in stem cell self-renewal across both the animal and plant kingdoms (Juliano et al., 2011).

Although the best understood role for PIWI genes is in the germline, mammalian PIWI homologs are also expressed in a range of somatic tissues. Recent studies suggest that their role is not limited to germ cells, in fact non-gonadal somatic roles have been reported in stem cell self-renewal, axon regeneration and memory formation (Ross et al., 2014; Jeong et al., 2021). However, reports on the expression of canonical piRNAs and the functional relevance of PIWI proteins beyond gonadal tissues are at times conflicting. In this respect, we have recently reported that a large part of the so-called somatic piRNAs, which have been described in non-germinal tissues, derive from non-coding RNAs which do not bear the specific molecular properties of canonical piRNAs (Tosar et al., 2018). We have introduced the concept of *miscellaneous*-piRNAs aiming to distinguish between canonical piRNAs and other sncRNAs that are circumstantially associated with PIWI proteins in somatic cells. *miscellaneous*-piRNAs include RNAs that, without being encoded in the canonical “piRNA clusters” at the genomic level (and therefore, not corresponding to the classification of piRNAs in the strict sense), could be associated with PIWI proteins in specific contexts; mediating their interaction with other proteins or, more likely, with other transcripts or the genome (Tosar et al., 2021). Additionally, we proposed that, PIWI proteins are broadly expressed and carried out functions beyond germline maintenance in their ancestral state (Horjales et al., 2023). Indeed, new functions for the piRNAs machinery are constantly being described in well-known model organisms such as *Drosophila*, where this machinery was first reported. One example is the modulation of developmental genes through translation initiation regulation by PIWI members in *Drosophila* and mice spermatogenesis (Rojas-Ríos and Simonelig, 2018; Dai et al., 2019; Ramat et al., 2020). These discoveries led to the concept of the PIWI/piRNAs pathway as a regulator of gene expression beyond transposons biology in somatic tissues (Liu et al., 2019).

Recently, the PIWI machinery appeared as a previously undescribed pathway in the initiation and progression of cancer. In fact, aberrant overexpression of PIWI proteins in somatic contexts has been shown in all cancer cell lines where they have been sought out. Furthermore, several studies showed piRNAs acting as oncogenes or tumor suppressors in cancer cells (Reviewed in (Liu et al., 2019)). Despite the challenging arguments against the somatic functions of piRNAs, dysregulation of the piRNAs machinery as a driver of human disease cannot be excluded.

### 1.2 PIWIL4 in acute myeloid leukemia

As mentioned above, recent studies have identified abnormal expression of components of the PIWI pathway in various cancer types, implicating its potential role in oncogenesis and tumor progression. Specifically, Bamezai *et al.* demonstrated that aberrant overexpression of PIWIL4 is essential for Acute Myeloid Leukemia (AML) development. AML is a severe hematologic malignancy driven by leukemia stem cells (LSCs) characterized by the uncontrolled proliferation and impaired differentiation of myeloid progenitors (Lapidot et al., 1994). Understanding the molecular mechanisms underlying these processes is crucial for developing targeted therapies.

Gene expression analyses revealed that, given its high expression among cancers studied and consistent overexpression in most AML patients, PIWIL4 could be a potential vulnerability that could be exploited as a therapeutic target in AML (Bamezai et al., 2023). In their study, Bamezai *et al.*, showed that PIWIL4 depletion using short hairpin RNA or guide RNA significantly reduced colony-forming ability and proliferation in AML cell lines and patient samples. Notably, this depletion impaired AML LSCs function, resulting in reduced engraftment in xenografts and delayed leukemia onset in mice. Furthermore, limiting dilution transplantation assays showed a marked reduction in LSC frequency upon PIWIL4 depletion. Furthermore, cell cycle analysis indicated increased DNA damage accumulation and apoptosis, highlighting the crucial role of PIWIL4 in AML LSCs, their progeny and disease progression.

In contrast to the demonstrated LSCs-specific dependency upon PIWIL4, PIWIL4 does not seem essential for the long-term repopulation capacity of healthy hematopoietic stem cells (HSPCs). While its expression in healthy HSPCs is lower compared to AML cells, it is still relatively high compared to most other tissues. Depleting PIWIL4 in healthy HSPCs did not affect colony formation, differentiation, engraftment in NSG mice, lineage specification nor did it induce apoptosis (Bamezai et al., 2023). These results suggest that PIWIL4 does not play a significant role in healthy HSPCs or normal hematopoiesis.

Bamezai *et al.*, additionally demonstrated that PIWIL4 plays a crucial role in preventing the accumulation of R-loops on specific genes associated with AML and LSCs. Depletion of PIWIL4 may interrupt transcription of genes crucial for LSC function and AML growth, as revealed by the overlap between PIWIL4 RNA targets and regions of R-loop enrichment and nascent transcription in AML cells.

Surprisingly, in AML cells, PIWIL4 binds to a small number of known piRNAs. However, it largely interacts with mRNAs corresponding to protein-coding genomic regions and enhancers enriched for cancer-associated and human myeloid progenitor gene signatures. As previously reported by our group, this is consistent with the absence of canonical piRNAs in these contexts (Tosar et al., 2021). According to Bamezai *et al.*, PIWIL4 appears to resolve R-loop structures by binding to RNA/DNA hybrids instead of fulfilling its canonical role by binding to piRNAs.

All in all, the studies discussed above:1) provide further evidence of the “non-canonical” function of PIWI pathways acting as gene expression regulators beyond the germline, 2) reveal that, while PIWIL4 executes its role of guarding of genomic stability in LSCs in a piRNA-independent manner it does not seem to impact normal hematopoiesis and 3) uncover a potential therapeutic avenue for the treatment of AML by targeting PIWIL4 in LSCs.

### 1.3 Newfound significance of PIWIL4 in preserving the stemness of leukemia cells?

**FIGURE 1:**
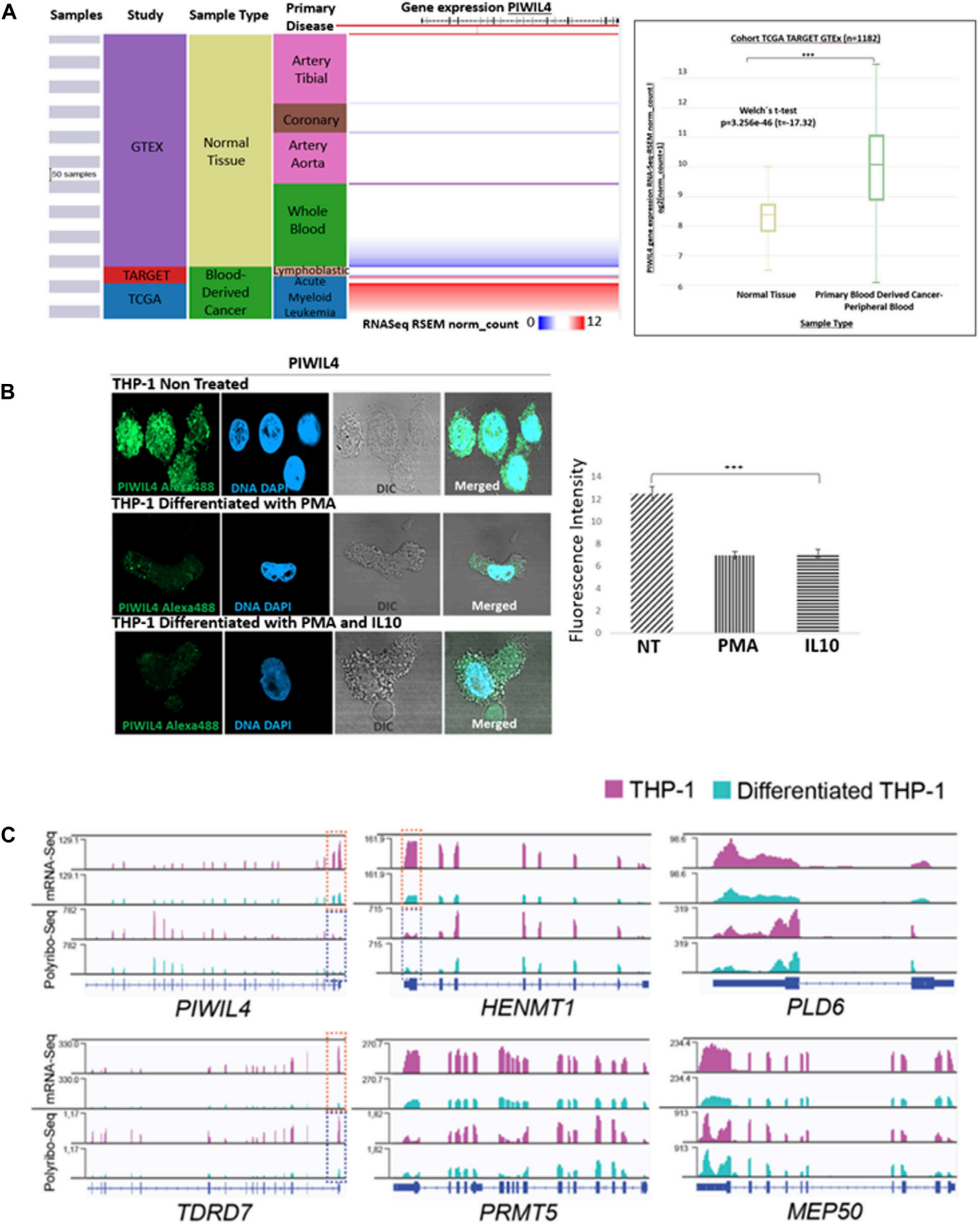
PIWIL4 is overexpressed in blood samples from AML patients and its expression is downregulated following differentiation in the THP-1 (AML-M5) model. **(A)**. Visual spreadsheet obtained from https://ucscxena.github.io for PIWIL4 in normal colon tissue (GTEX), TARGET and TCGA cancer databases. Left panel: UCSC Xena visual spreadsheet for *PIWIL4* mRNA in normal and primary tumor-derived samples. Right Panel: *PIWIL4* levels in blood cancer samples from TCGA/TARGET and normal blood from GTEx (*p* ≤ 0.0001). **(B)**. Left Panel: Indirect immunofluorescence of PIWIL4 in THP-1 monocytes and THP-1-derived macrophages generated by exposure to PMA or/and Il-10. PIWIL4 (green) is expressed at high levels in the undifferentiated THP-1 monocytes. Right Panel: Fluorescence signal quantification. **(C)**. Expression of PIWI pathway genes in undifferentiated and differentiated THP-1 cells by mRNA-Seq and Polyribosome Sequencing. Coverage plots of reads per kilobase per million reads (RPKM) for mRNA-Seq (top) and Polyribo-Seq (bottom). Overall, gene expression levels decrease following differentiation of THP-1 cells. A region of key transcripts is highlighted within a doted box (mRNA in orange, polyribosomal mRNA in blue) for visualization purposes.

To further substantiate the findings of Bamezai et al., we established PIWIL4 expression in normal vs*.* cancer blood-derived samples from patients from GTEx, TARGET and TCGA projects using Xena Browser. As anticipated, PIWIL4 exhibited overexpression in blood samples derived from cancer patients compared to their normal counterparts (Figure 1A).

The main aim of this perspective is to bring into focus PIWIL4 as a potential regulator of myeloid cell differentiation in the context of AML, a malignancy characterized by accumulation of immature or undifferentiated progenitor cells as a consequence of differentiation blockage. As a first approach, we investigated the expression of PIWIL4 in the monocytic cell line THP-1, a widely used model of human AML (AML-M5) that retained its differentiation capacity into THP-1-derived macrophages by exposure to specific stimulus (Mohd Yasin et al., 2022).

As expected, immunofluorescence staining analysis revealed that PIWIL4 is robustly expressed in monocytic (undifferentiated) THP-1 cells. However, upon differentiation into THP-1-derived macrophages induced by stimulation with phorbol 12-myristate 13-acetate (PMA) and IL-10 (Baxter et al., 2020), we observed a significant downregulation of PIWIL4 expression (Figure 1B).

In line with our immunofluorescence staining analysis, exploration of our recently published mRNA- and Polyribo-seq datasets generated in THP-1 monocytes and THP-1- derived macrophages (Pinello et al., 2024) revealed a marked decrease in PIWIL4 expression in macrophages compared to monocytes (Figure 1C). Furthermore, a number of key PIWI-pathway genes including HENMT1,TDRD7, PLD6, PRMT5 and MEP50 (Li et al., 2020) are highly transcribed (mRNA-Seq) and actively translated (Polyribo-Seq) in these THP-1 monocytes. Notably, THP-1-derived macrophages expressed lower levels of PIWIL4, HENMT1 and other components of the pathway than undifferentiated cells (Figure 1C).

Overall, we confirmed that PIWIL4 is overexpressed in leukemic samples and in THP-1 as reported by Bamezai *et al* (Bamezai et al., 2023) and we report that the expression levels of PIWIL4 and other key genes within the PIWI pathway are downregulated following differentiation.

## 2 Discussion

This perspective shapes upon recently published evidence pointing to PIWI proteins as key players in leukemia pathogenesis (Wang et al., 2015; Bamezai et al., 2023; Ding et al., 2023). Despite the growing interest in PIWIL4’s role in cancer its expression dynamics during myeloid cell differentiation remain unclear. In our study, we examined PIWIL4 expression before and after differentiation of THP-1 monocytes into macrophages to contribute to elucidate the potential implications of PIWIL4 expression dynamics in the context of myeloid differentiation and function. We have showed that PIWIL4 mRNA and protein levels were significantly reduced in THP-1-derived macrophages compared to monocytic THP-1 cells.

Previous reports from our group have uncovered a role for PIWIL1 in controlling cell cycle progression in Colorectal Cancer (CRC). Further, we reported that PIWIL1 expression is lost during differentiation suggesting that somatic expression of PIWI proteins could be restricted to stem/cancer stem cells (Silva et al., 2024). The observation that differentiation correlates with silencing of key PIWI pathway genes both in non-terminally differentiated CRC (Silva et al., 2024) and AML monocytes (Figure 1) is remarkable. Notably, HENMT1, the enzyme responsible for modifying the 3′-end of piRNAs, has been reported as one of the most highly dysregulated RNA modifying proteins in cancer including AML and colon adenocarcinoma (Begik et al., 2020). These observations together with the work of Bamezai *et a*l, add to the growing body of evidence showing that PIWI pathway, beyond its well-established roles in gametogenesis, is vital for the survival and perhaps modulation of differentiation of leukemia stem cells. Importantly, Bamezai *et a*l propose a mechanism by which PIWIL4 binds RNA from genes associated with LSC maintenance factors known to be sites of R-loop enrichment in AML cells. Whether this mechanism is AML specific or if it is also acting in other cancer types and/or their healthy counterparts remains to be answered. However, it is important to highlight that more studies are needed to understand these findings on the role of the PIWI pathway in affecting AML stemness.

**FIGURE 2:**
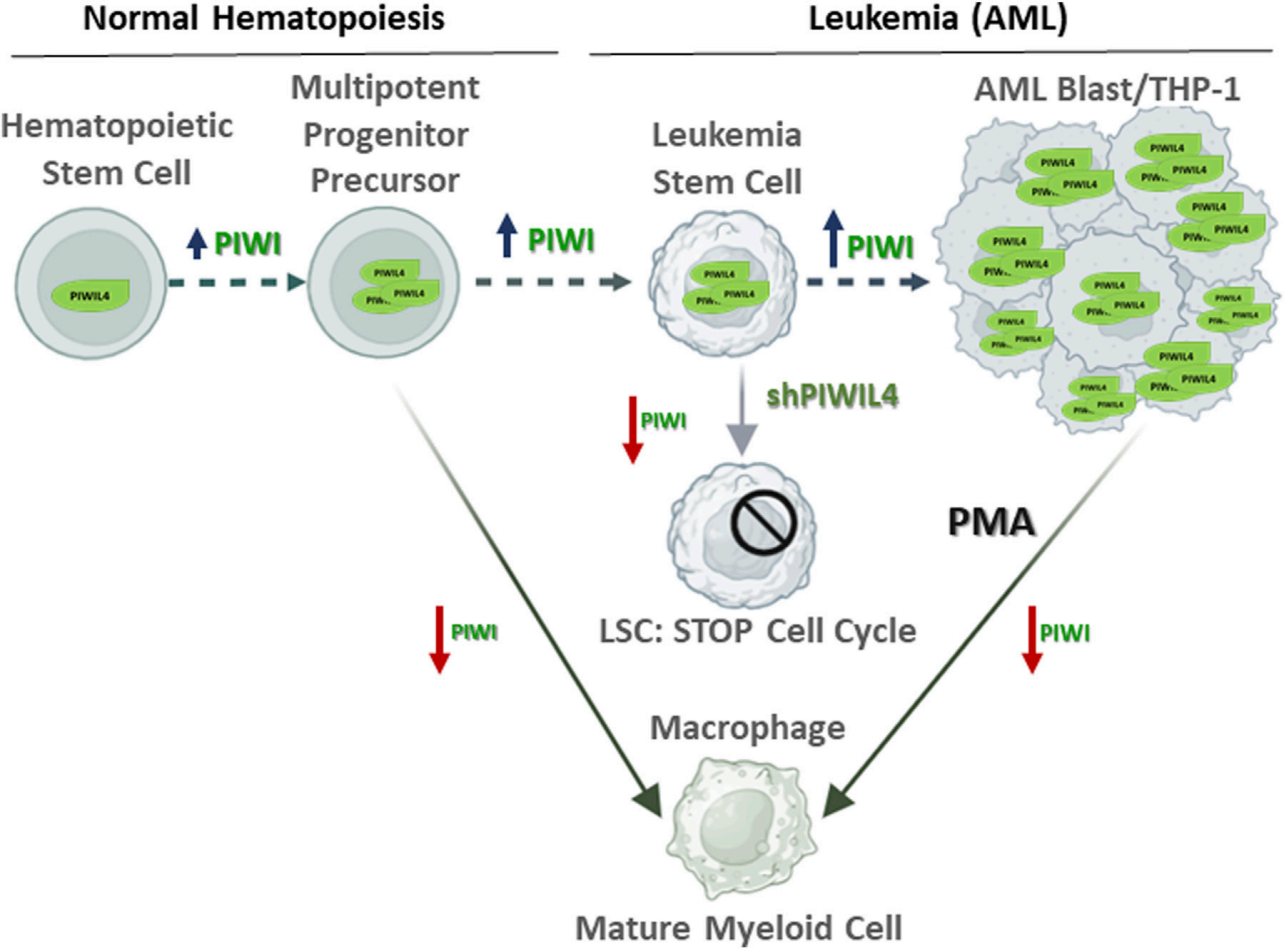
Proposed model highlighting the role of PIWIL4 maintaining the stemness state of Leukemia Stem Cells (LSCs) in acute myeloid leukemia (AML). In normal hematopoietic stem cells (HSCs), in line with its non-essential role in healthy hematopoiesis, PIWIL4 expression remains relatively low. In contrast, PIWIL4 is overexpressed in AML where it contributes to sustaining AML LSCs stemness. Aberrant PIWIL4 overexpression leads to uncontrolled proliferation and impaired differentiation of myeloid progenitors a hallmark of AML. Studies by Bamezai *et al.* showed that PIWIL4 depletion prompts cell cycle arrest. Similarly, THP-1 cells exposed to the differentiating agent PMA undergo cell cycle arrest prior initiating differentiation into macrophages and downregulate PIWIL4 expression. We propose differentiation-associated modulation of PIWIL4 in myeloid cells as potential therapeutic target in AML. Red arrows: PIWI overexpression; blue arrows: PIWI low expression.

Here, we present an additional perspective on the potential role of PIWIL4 contributing to the differentiation status of AML and the emergence of LSCs responsible for leukemia development and maintenance. While Bamezai *et al.* described the overexpression of PIWIL4 in myeloid-derived cancers and its potential contribution to the malignant phenotype, our observations suggest that PIWIL4 may be also involved in cellular differentiation. We observed that PIWIL4 expression in the THP-1 monocytic cell line significantly decreases when differentiated into macrophages, suggesting that PIWI proteins could potentially have a role in maintaining the undifferentiated state of myeloid cells. Our findings, that positively correlate with PIWIL4 overexpression observed in various myeloid-derived cancers as reported by Bamezai *et al.*, indicate that, besides maintaining LSCs stemness, PIWIL4 could be also involved in modulating cellular differentiation status as represented in Figure 2. However, the question remains open: What is the relevance for PIWI pathway overexpression in AML for disease initiation, progression and treatment?

Another important point to consider is that while overexpression of PIWI-pathway proteins in cancer is well characterized, whether these proteins work in association with piRNAs in somatic contexts remains controversial (Tosar et al., 2018; 2021). Collectively, these reports ((Tosar et al., 2018; 2021)) are in line with Bamezai *et a*l. findings in the sense that AML cells owe their dependency on PIWIL4 to resolve R-loops in a non-piRNA-dependent manner and therefore executing non-canonical function for PIWI proteins. Finally, we propose that efforts should be directed towards elucidating the functions of the PIWI pathway not only in oncogenesis but also in the context of basic cellular processes such as differentiation with the aim to identify novel biomarkers and therapeutic targets for the treatment of AML.

## Data Availability

The original contributions presented in the study are included in the article/supplementary material, further inquiries can be directed to the corresponding author.
